# A proof of concept: digital diary using 24-hour monitoring using wearable device for patients with Parkinson’s disease in nursing homes

**DOI:** 10.3389/fneur.2024.1356042

**Published:** 2024-04-10

**Authors:** Hikaru Kamo, Genko Oyama, Yui Yamasaki, Tomohiro Nagayama, Ryotatsu Nawashiro, Nobutaka Hattori

**Affiliations:** ^1^Department of Neurology, Juntendo University School of Medicine, Tokyo, Japan; ^2^Department of Home Medical Care System, Based on Information and Communication Technology, Juntendo University Graduate School of Medicine, Tokyo, Japan; ^3^Department of Drug Development for Parkinson's Disease, Juntendo University Graduate School of Medicine, Tokyo, Japan; ^4^Department of PRO-Based Integrated Data Analysis in Neurological Disorders, Juntendo University Graduate School of Medicine, Tokyo, Japan; ^5^Research and Therapeutics for Movement Disorders, Juntendo University Graduate School of Medicine, Tokyo, Japan; ^6^Sunwels Company Limited, Chiyoda-ku, Tokyo, Japan; ^7^Research Institute of Disease of Old Age, Graduate School of Medicine, Juntendo University, Tokyo, Japan; ^8^Neurodegenerative Disorders Collaborative Laboratory, RIKEN Center for Brain Science, Saitama, Japan

**Keywords:** Parkinson’s disease, wearable device, physiological biomarker, heart rate, wearing off, dyskinesia

## Abstract

**Introduction:**

In the advanced stages of Parkinson’s disease (PD), motor complications such as wearing-off and dyskinesia are problematic and vary daily. These symptoms need to be monitored precisely to provide adequate care for patients with advanced PD.

**Methods:**

This study used wearable devices to explore biomarkers for motor complications by measuring multiple biomarkers in patients with PD residing in facilities and combining them with lifestyle and clinical assessments. Data on the pulse rate and activity index (metabolic equivalents) were collected from 12 patients over 30 days.

**Results:**

The pulse rate and activity index during the *off-* and *on-*periods and *dyskinesia* were analyzed for two participants; the pulse rate and activity index did not show any particular trend in each participant; however, the pulse rate/activity index was significantly greater in the *off*-state compared to that in the *dyskinesia* and *on*-states, and this index in the *dyskinesia* state was significantly greater than that in the *on*-state in both participants.

**Conclusion:**

These results suggest the pulse rate and activity index combination would be a useful indicator of wearing-off and dyskinesia and that biometric information from wearable devices may function as a digital diary. Accumulating more cases and collecting additional data are necessary to verify our findings.

## Background

Parkinson’s disease (PD) is a progressive neurodegenerative disease that can be effectively treated with levodopa during its early stages (1). However, treatment becomes challenging as the disease advances because of the emergence of motor complications such as wearing-off and levodopa-induced dyskinesia (2). In cases with medically refractory motor complications, device-aided therapies are options, including deep brain stimulation (DBS), levodopa-carbidopa intestinal gel infusion, and subcutaneous drug infusions. Even after device-aided therapies, motor complications may re-emerge over time (3). Given these challenges, continuous and objective motor complication monitoring is essential to manage PD symptoms effectively.

Wearable devices have been studied extensively for monitoring Parkinsonian symptoms (4). However, differentiating between *on* and *off* states using accelerometer-based monitoring is technically challenging when a patient is at rest. Active monitoring requires patients to perform specific tasks and can accurately detect the *off*-state (5). However, this method can be intrusive; maintaining adherence to these tasks presents a significant challenge. Consequently, a practical and patient-friendly method for detecting the *off*-state using passive monitoring with a wearable device has yet to be implemented effectively in clinical settings.

This study used multiple sensor technologies to explore biomarkers capable of detecting the *off*-state. Residents in elderly care facilities were monitored for 24 continuous hours to investigate the relationship between Parkinsonian symptoms and physiological biomarkers gathered from wearable devices.

## Methods

### Participants

The study included residents in elderly care facilities specializing in PD (PD houses, Sunwels Ltd., Tokyo). Inclusion criteria were (1) ≥20 years of age, (2) residents of a PD house (Imajuku, Nishimiyanosawa, Hakusan, and Arita), (3) previously diagnosed with PD, and (4) who provided written consent. Patients who had difficulty wearing the wearable device were excluded.

### Study protocol

The study was conducted from March 2022 to June 2022. The participants wore a three-axis multisensory wearable device (iAide2, TOKAI corp., Gifu, Japan) on their left or right wrist for 24 h (except during charging) for >30 days. This device tracked the pulse rate and activity index. The activity index is calculated using a proprietary algorithm corresponding to metabolic equivalents (METs) (6). If fully charged, the battery life of the wearable device (iAide2) is 8 days. The participants re-charged the battery for only a few minutes each day, such as during bath time. In addition, the Nemuri-SCAN (Paramount Bed, Tokyo, Japan), a sensor sheet placed beneath the mattress, monitored their pulse rate during sleep and detected instances of getting out of bed. Data were collected every 20 s; the pulse rate and activity index were averaged every minute daily. The facility care staff recorded the timing of meals, medications, rehabilitation, and subjective *off*-state, *on*-state, and *dyskinesia* times. The *on*-state was defined as any period other than the *off*, *dyskinesia*, and sleep times, which the Nemuri-Scan detected. Rehabilitation was provided 0–7 times per week, 0–3 times per day, depending on the symptoms. Rehabilitation could not be performed for a period during the COVID-19 outbreak.

This study was approved by the Juntendo University Ethics Committee (#M20-0294-M01). All participants provided written informed consent.

### Statistical analysis

Statistical analysis was performed using JMP software (Ver 16, SAS, United States). The main parameters analyzed were pulse rate and activity index. Because of the non-parametric nature of the data and the requirement for multiple comparison procedures, the Steel-Dwass test was used. The Kruskal–Wallis test was used to compare the pulse rate coefficient of variance and average pulse rate across different patients. Furthermore, the Pearson product–moment correlation coefficient was calculated to evaluate the relationship between pulse rate measurements from the Nemuri-SCAN and the iAide-2 devices. In all statistical tests, *p* < 0.05 was statistically significant.

## Results

### Participants characteristics

The study included 12 participants with PD (6 males and 6 females) in four PD house facilities: Imajuku (*n* = 1), Nishimiyanosawa (*n* = 1), Hakusan (*n* = 4), and Arita (*n* = 3). The age at consent was 76.5 ± 8.4 years, disease duration was 9.7 ± 5.5 years, Hoehn and Yahr stage was 3.2 ± 0.4 at *on* and 3.9 ± 0.5 at *off*, Mini-Mental State Examination score was 27.1 ± 3.8, and Functional Independence Measure was 90.8 ± 11.2. The total levodopa equivalent daily dose was 645.4 ± 360.1, calculated using standard conversion factors (7).

### Data collected by wearable devices

**Figure 1:**
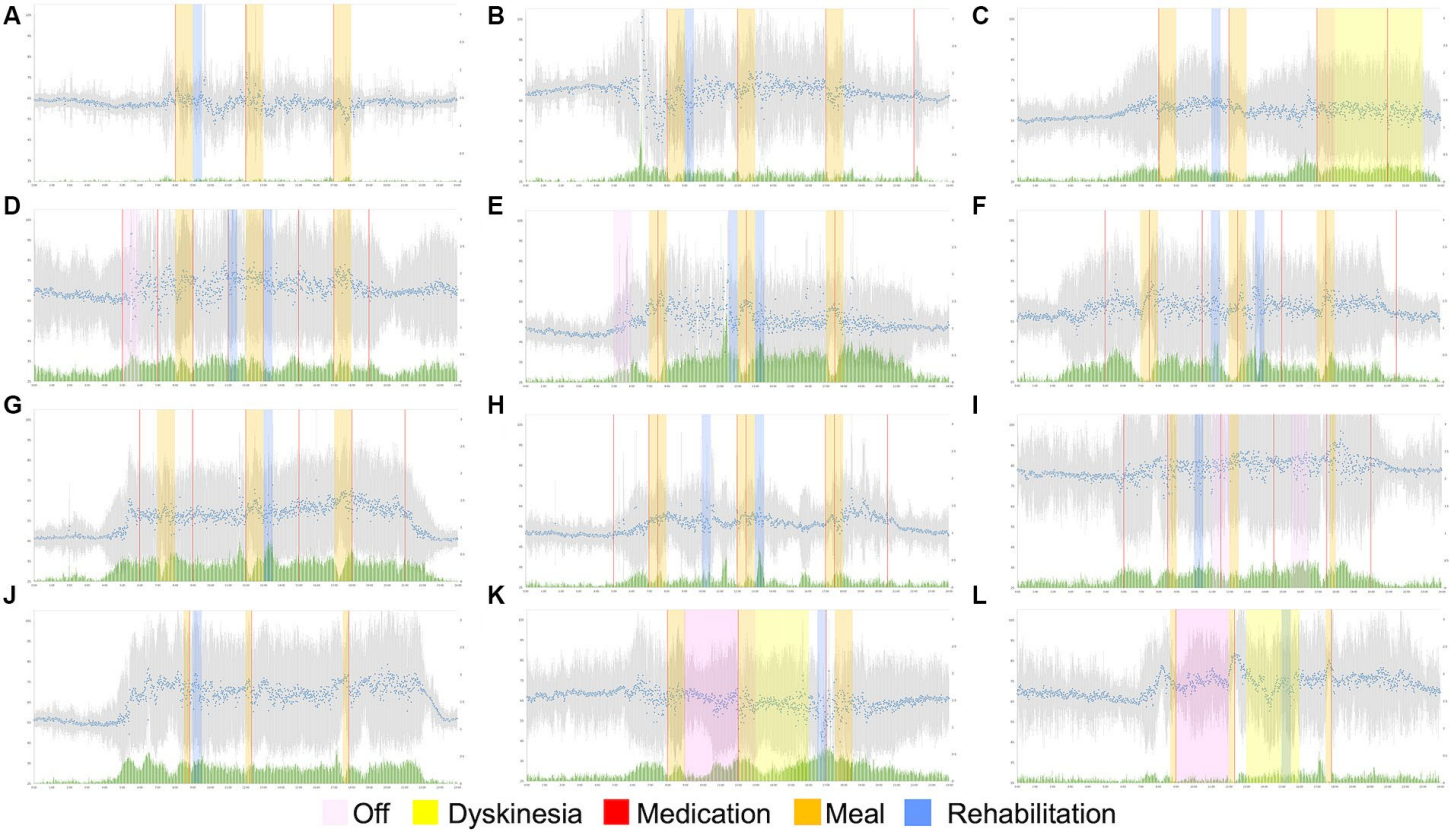
Diurnal variation in pulse rate/activity and subjective symptoms of PD for each participant. The 30-day average pulse rate and activity level monitored for 24 h using a wearable device are plotted. The horizontal axis represents time, and the vertical axis represents either the pulse rate or activity level. The facility staff recorded medications, meals, and rehabilitation times. The time *off* or with dyskinesia is highlighted based on the patient’s subjective symptoms. Patients 1–12 are represented in the figure by panels **(A–L)**.

Twelve participants completed the data collection over 30 days. Figure 1 shows the 30-day average pulse rate and activity index measured for 24 h using a wearable device. Among the 12 participants, 3 had wearing-off (Participants #4, #5, and #9), 1 had dyskinesia (Participant #3), and 2 had both wearing-off and dyskinesia periods (Participants #11 and #12).

### Exploration of a digital biomarker for wearing-off and dyskinesia

**Figure 2:**
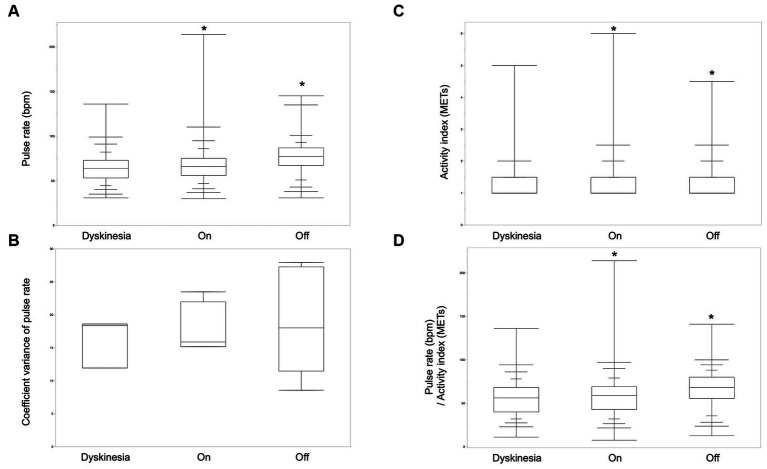
Biological signals of wearing-off and dyskinesia. **(A)** The pulse rates were significantly greater in the order of the *off-*state, *on-*state, and *dyskinesia* in six participants who had *off-* and/or dyskinesia periods (Steel-Dwass test, *p* < 0.001). **(B)** The pulse-rate coefficient variance was not significantly different between *dyskinesia*, the *on-period,* and the *off-*period (Kruskal–Wallis test, *p* = 0.69). **(C)** The activity index was significantly greater in the order of the *on*-state, *dyskinesia*, and the *off*-state (Steel-Dwass test, *p* < 0.001). **(D)** Pulse rate/activity index (METs) per minute was significantly greater in the order of the *off*-state, the *on*-state, and, *dyskinesia* (Steel-Dwass test, *p* < 0.001).

The pulse rate and activity index derived from the wearable device during the *off-*period, the *on-*period, and *dyskinesia*, as defined by the symptom diary, were analyzed to explore digital biomarkers for wearing-off and dyskinesia in patients with PD. The analysis included 6 participants with PD who exhibited one or both of these symptoms. Pulse rates were significantly greater in the *off*-state than in the *on* and *dyskinesia* states. Moreover, the pulse rates in the *on*-state were also significantly greater than that in the *dyskinesia* state (Steel-Dwass test, *p* < 0.001). The pulse rate coefficient of variance was not significantly different between dyskinesia and the *on-* and *off-*states (Kruskal–Wallis test, *p* = 0.69). The activity index was significantly greater in the order of the *on*-state, *dyskinesia*, and the *off*-state (Steel-Dwass test, *p* < 0.001). The pulse rate/activity index (METs) per minute was significantly greater in the order of the *off*-state, the *on*-state, and *dyskinesia* (Steel-Dwass test, *p* < 0.001; Figure 2).

The average pulse rate differed among the patients (Kruskal–Wallis test, *p* < 0.001). Pulse rates were measured using multiple devices to ensure reliability, with significant correlations between the one-minute pulse rates measured simultaneously by Nemuri-SCAN and iAide-2 (Pearson product–moment correlation coefficient, r = 0.51, *p* < 0.001). This cross-verification reinforces the potential of these measures as digital biomarkers of wearing-off and dyskinesia in patients with PD.

**Figure 3:**
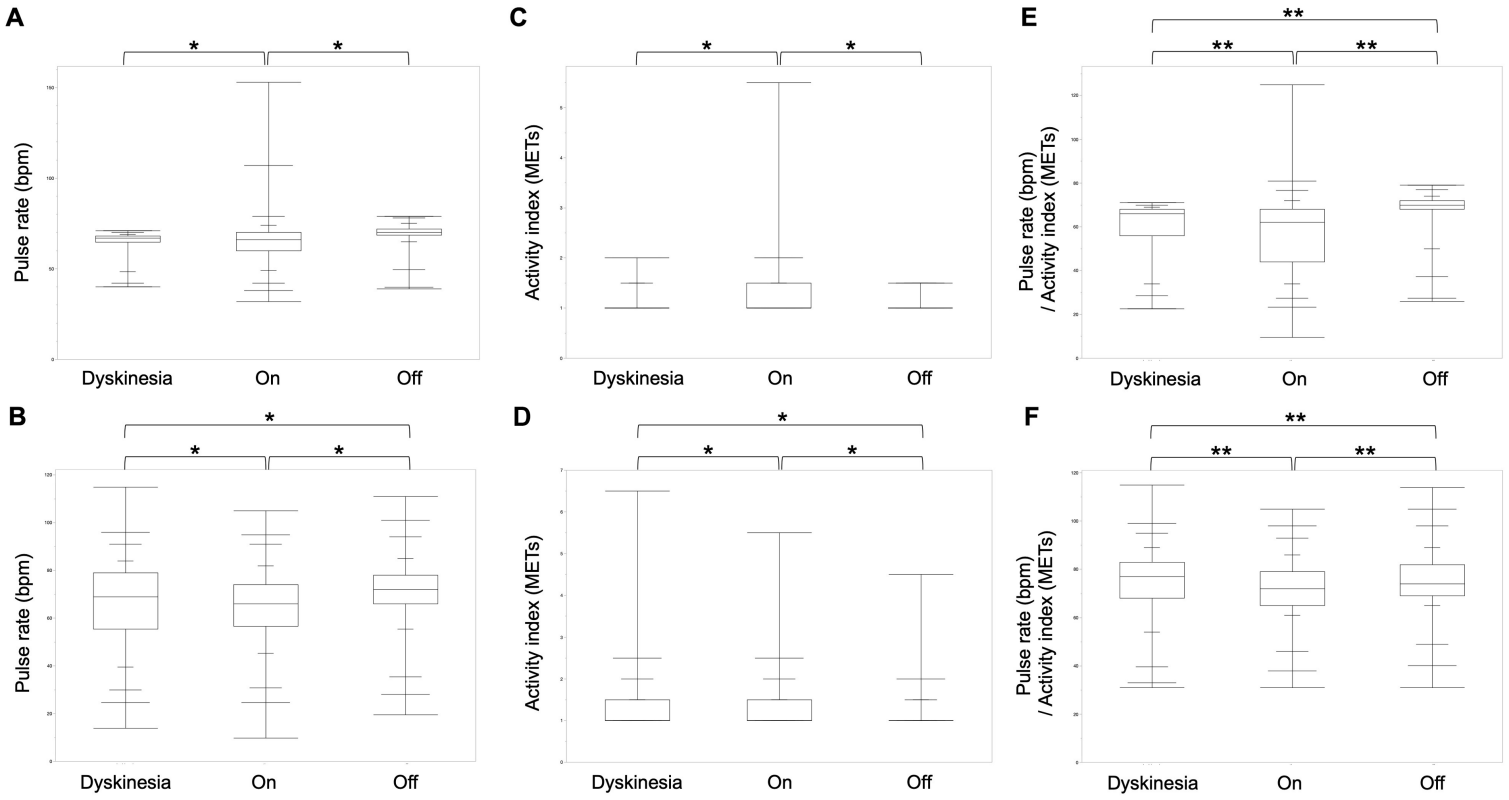
Comparison of the pulse rate, activity index, and pulse rate/activity index for each condition in two participants. **(A)** The pulse rate was significantly higher during the *off*-state, but not significantly different between *dyskinesia* and the *on-*state for participant 11 (Steel-Dwass test, *p* < 0.001). **(B)** The pulse rate was significantly higher in the order of the *off*-state, *dyskinesia*, and the *on*-state for participant 12. **(C)** The activity index was significantly higher in the *on*-state, but not significantly different between dyskinesia and the *off-*state for participant 11 (Steel-Dwass test, *p* < 0.001). **(D)** The activity index was significantly higher in the order of *dyskinesia*, the *on*-state, and the *off*-state for participant 12 (Steel-Dwass test, *p* < 0.001). **(E,F)** In both participants, the pulse rate/activity index (METs) was significantly greater in the order of the *off*-state, *dyskinesia*, and the *on*-state (Steel-Dwass test, *p* < 0.005).

We compared the pulse rate and activity index in two participants with *dyskinesia* and *off* periods for a more detailed analysis (Table 1). In both participants, the pulse rate was significantly higher in *off*-state than *dyskinesia*, and the *on*-state, and in comparing *dyskinesia*, and the *on*-state, the pulse rate was significantly higher in *dyskinesia* state than *on*-state for participant 12 (Steel-Dwass test, *p* < 0.001), while it was significantly higher during the *off*-state, but not significantly different between *dyskinesia* and the *on*-state for participant 11 (Steel-Dwass test, *p* < 0.001). The activity index (METs) was significantly higher in the *on*-state, but not significantly different between dyskinesia and the *off*-state for participant 11 (Steel-Dwass test, *p* < 0.001); it was significantly higher in the order of *dyskinesia*, the *on*-state, and the *off*-state for participant 12 (Steel-Dwass test, *p* < 0.001). In both participants, the pulse rate/activity index (METs) was significantly greater in the order of the *off*-state, *dyskinesia*, and the *on*-state (Steel-Dwass test, *p* < 0.005; Figure 3).

**Table 1:** The pulse rate, activity index, and pulse rate/activity index in the in participants with both dyskinesia and off periods.

		Pt 11	Pt 12
		Median [IQR]	Median [IQR]
Pulse rate (bpm)	Dyskinesia	67.0 [64.8–68.0]	77.0 [68.0–83.0]
	On	66.0 [60.0–70.0]	72.0^*^ [65.0–79.0]
	Off	70.0^*^ [68.5–72.0]	74.0^*^ [69.0–82.0]
Activity index (METs)	Dyskinesia	1.0 [1.0]	1.0 [1.0–1.5]
	On	1.0^*^ [1.0–1.5]	1.0^*^ [1.0–1.5]
	Off	1.0 [1.0]	1.0^*^ [1.0]
Pulse rate (bpm)/activity index (METs)	Dyskinesia	66.0 [56.0–68.0]	69.0 [53.3–79.0]
	On	62.0^**^ [44.0–68.0]	66.0^**^ [56.7–74.0]
	Off	70.0^**^ [68.0–72.0]	72.0^**^ [66.0–78.0]

^*^The pulse rate was significantly high during the *off-*state compared to that during the *on-*state or *dyskinesia* in participant 11 and significantly higher in the order of the *off*-state, *dyskinesia*, and the *on*-state in participant 12 (Steel-Dwass test, *p* < 0.001). The activity index was significantly higher in the *on*-state, but not significantly different between dyskinesia and the *off-*state in participant 11; it was significantly higher in the order of *dyskinesia*, the *on*-state, and the *off*-state in participant 12 (Steel-Dwass test, *p* < 0.001). ^**^In both participants, the pulse rate/activity index (METs) was significantly greater in the order of the *off*-state, *dyskinesia*, and the *on*-state (Steel-Dwass test, *p* < 0.005).

## Discussion

This study demonstrated that multiple wearable devices could monitor diurnal changes in patients with PD. It is difficult to distinguish the conditions of patients with PD using only the pulse rate or activity index. However, the pulse rate per activity index was significantly greater in the order of the *off-*state, *dyskinesia*, and the *on-*state. These results indicate that the simultaneous monitoring of biometric and motion information using an accelerometer and wearable devices provides meaningful information for the *off*-state and *dyskinesia*. This monitoring may function as a digital diary instead of a paper-based diary for evaluating a patient’s condition. Recent advances in digital and telecommunication technologies have brought innovation to medicine. The integration of artificial intelligence, next-generation communication networks, and Internet of Things is anticipated to facilitate the management of larger datasets and enable personalized and tailor-made medical care in the future (8).

Traditionally, PD treatment relies on both patient-reported conditions and physical examination findings. However, motor complications can be prominent in the advanced stages of PD; a common finding is that some patients exhibit favorable conditions during medical examinations but experience discomfort in their daily lives. Another challenge is that some patients cannot distinguish between dyskinesia and tremor or report symptoms originating from conditions unrelated to PD. These complexities make accurately monitoring the specific PD state causing their discomfort challenging. Symptom diaries are the most commonly used method for documenting motor symptom fluctuations in daily life (9). However, because of their subjective nature, symptom diaries are often inconsistent with the physician’s diagnosis (10). Therefore, developing a tool for obtaining objective information from symptom diaries is an important and unmet need for both patients and clinicians.

Methods for collecting physiological biomarkers include surface electromyography, local field potentials from DBS leads implanted in basal ganglia, electrocorticography (ECoG), and mobile health (mHealth) devices. Surface electromyography is historically used to diagnose tremors and is a reliable biomarker (11, 12). However, the methods to detect bradykinesia and rigidity have not been established (13, 14), and distinguishing between immobility and the mobile-but-resting state is still difficult by using accelerometers or wearable devices. Beta-band activity (beta oscillation) of the local field potential (LFP) recorded in the subthalamic nucleus is a promising biomarker correlated with the severity of bradykinesia and rigidity; administration of levodopa or DBS stimulation decreases beta oscillations (15–18). However, the LFP in the subthalamic nucleus has not been established to correlate with tremors or dyskinesia (19–21) and requires invasive procedures. ECoG measures brain electrical activity by placing sheet electrodes on the cortical surface. In PD, phase-amplitude coupling detected using ECoG is elevated between beta and broadband gamma (50–150 Hz) over the motor cortex and correlates with immobility and rigidity (22). Furthermore, fine-tuned gamma oscillations (60–90 Hz) in the cortex have relatively high specificity for dyskinesia (21). Although it is less invasive than DBS, it requires the implantation of subdural grids, which risks bleeding and infection.

Health management increasingly uses mHealth devices, including smartphones and wearable devices. Smartphone applications are highly popular and have advantages such as inputting patient-reported outcomes. However, they are not always worn on a specific body part and rely on subjective patient reports (23). Wearable devices using accelerometers and gyroscopes have been studied to monitor PD symptoms (24). Wearable devices are superior at detecting tremors compared to LFP (12, 25) and are expected to be effective for detecting freezing of gait, bradykinesia, and dyskinesia (26–29). Passive monitoring using wearable devices equipped with accelerometers and gyroscopes faces challenges in accurately detecting bradykinesia. Differentiating between a patient’s voluntary rest period and the symptomatic *off*-state period is difficult (30, 31) and requires active monitoring, which requires patients to perform specific tasks. Although biological biomarkers for a single symptom of PD are being identified, a method to monitor all symptoms of PD with a single device has not been established; therefore, treatment focusing on only a single symptom of PD will not improve the quality of life.

This study hypothesized that combining multiple biological biomarkers obtained from wearable devices could overcome these problems. The results indicate that combining pulse rate and activity level might distinguish between three states, spontaneous resting, *off*, and dyskinesia, representing a novel proof of concept in monitoring the *off*-state in PD. Indeed, Powers et al. reported that an Apple Watch can use inertial sensors to detect tremors and dyskinesia (32) but not bradykinesia. Multiple factors such as emotion and autonomic nerve systems are thought to be involved in the higher pulse rate during the *off-*state than any other conditions. Patients with advanced PD have a lower skin temperature, increased sweating, and higher orthostatic blood pressure in the *off-*state compared to those in the *on-*state (33). This is thought to result from the presence of autonomic failure in patients with PD (34, 35). However, because the pulse rate and other autonomic indicators are greatly influenced by emotions and behavior, it is considered difficult to use the autonomic nervous system alone as a biomarker for wearing-off (36). Anxiety and depression, major non-motor disorders, are induced during the *off-*state and may also affect the pulse rate elevation (37). Low physical activity during the *off-*period is due to bradykinesia, which is difficult to distinguish from rest, making it more challenging to determine the status of patients with PD by activity monitoring alone. To the best of our knowledge, previous studies have only investigated the representation of motor symptoms in patients with PD by using the autonomic nervous system alone or activity monitoring alone. Herein, we have shown that it is possible to describe the condition of patients with PD by combining different indices of activity monitoring and the pulse rate.

The limitations of this study include the small number of participants, its observational nature, and the possibility of underestimating exercise and stress. Furthermore, the data were collected in a homogenized environment, the same group of facilities specializing in the care of patients with PD, and clinical data were collected daily by the facilities’ staff; therefore, our results cannot be generalized to real-world situations. The wearable device was worn on any arm in this study; thus, the activity index may have changed during the movement depending on whether or not the device was worn on the dominant hand. Further analysis is needed to identify a biomarker correlating spontaneous movement with a dyskinesia state and to confirm our concept of developing digital diaries using biological biomarkers. Moreover, the decision time to distinguish the condition of the patient needs to be set for practical use. Since irregular values may be obtained due to sudden activity or stress factors, a relatively longer judgment time may more accurately represent the condition. Alternatively, real-time judgment is desired for practical application. In future research, it is necessary to search for a judgment time that balances accuracy and practicality.

This study demonstrated that the combination of biological signals and movement data obtained from wearable devices has the potential to be a biomarker for the fluctuating symptoms of PD. This approach could be instrumental in developing a digital daily tailored to PD management. However, further studies are required to validate these hypotheses. Such studies should involve collecting more comprehensive data and applying a machine-learning approach to develop a robust model that accurately corresponds to the precise condition of patients with PD.

## Data availability statement

The raw data supporting the conclusions of this article will be made available by the authors, without undue reservation.

## Ethics statement

The studies involving humans were approved by Juntendo University Ethics Committee (#M20-0294-M01). The studies were conducted in accordance with the local legislation and institutional requirements. The participants provided their written informed consent to participate in this study.

## Author contributions

HK: Writing – review & editing, Writing – original draft, Project administration, Methodology, Formal analysis, Data curation, Conceptualization. GO: Writing – review & editing, Supervision, Methodology, Investigation, Conceptualization. YY: Writing – review & editing, Methodology, Investigation, Funding acquisition, Conceptualization. TN: Writing – review & editing, Funding acquisition. RN: Writing – review & editing, Funding acquisition. NH: Writing – review & editing.
